# Enhanced Flocculation of Oil Sands Mature Fine Tailings Using Hydrophobically Modified Polyacrylamide Copolymers

**DOI:** 10.1002/gch2.201700135

**Published:** 2018-02-23

**Authors:** Rhys Hripko, Vahid Vajihinejad, Fernanda LopesMotta, João B. P. Soares

**Affiliations:** ^1^ Department of Chemical and Materials Engineering University of Alberta 9211 116 St Edmonton Alberta T6G 1H9 Canada

**Keywords:** dewatering, flocculation, oil sands tailings, polyacrylamide‐poly(ethylene oxide methyl ether methacrylate) copolymers, statistical design

## Abstract

Hydrophobically modified acrylamide copolymers dewater oil sands tailings more effectively than anionic polyacrylamide, but the root causes for this enhanced performance have not been investigated systematically. Polyacrylamide‐poly(ethylene oxide methyl ether methacrylate) copolymers with different comonomer compositions, hydrophobic chain lengths, and molecular weights to map out these effects systematically are synthetized. Through a statistical design of experiments, it is found out that all three variables above significantly affected flocculation performance and that certain combinations achieve optimal results. The effect of centrifugation on the flocculation and dewatering performance of these polymers is also investigated.

## 1 Introduction

There is a stigma surrounding the Alberta oil sands in terms of environmental hazards. One of the main contributors to this stigma is the generation of large volumes of waste stored in tailings ponds. Tailings ponds are composed of water, residual bitumen, fine mineral particles, and organic compounds, covering an area more than 175 km^2^ which is approximately the area that would be occupied by 20 million average sized cars.^1^ Masliyah et al. stated that this problem is getting worse—for every barrel of crude bitumen extracted, 3.3 m^3^ of tailings are discharged in the environment.^2^ This problem is not going away any time soon, since the negatively charged fine particles (mostly clays) suspended in the tailings prevent the suspension from settling.^3^ To wait many decades for the tailings ponds to consolidate naturally is unacceptable not only for the oil sands processing companies, but also for the Canadian society. Many researchers are trying to find solutions to this problem, and several are investigating novel water‐soluble polymers to achieve rapid flocculation and dewatering.^4^, ^5^, ^6^ Increasing the efficiency of tailings separation would be immediately beneficial to oil sands processing companies, as it would significantly lower both the volume of tailings creation and costs of operation.^6^

The industry has practiced several technologies to dewater and consolidate tailings. Thin lift and freeze‐thaw drying, centrifugation, and composite tailing are examples of some of these practices.^7^, ^8^ In thin lift drying, for example, a thin layer of polymer‐flocculated tailings is spread over a land area with small slope, and allowed to dewater until the remaining sediments are ready for land reclamation. The method is easy and relatively inexpensive, but requires a large land area. Another technology is composite tailing, where fluid fine tailings are mixed with sand and are flocculated using polymers or chemical additives such as gypsum. The sand helps flocs dewater more rapidly by making channels in the sediments, and it also strengthens the sediments. One drawback of this method is the use of large amounts of sand, which otherwise could be used to build storage dikes for tailings.^7^ Centrifugation is also a technology that may be used for the densification of sediments after flocculation, if run under conditions that are economically viable. Mikula et al. concluded that centrifugation of mature fine tailings (MFT) could drastically reduce the water loss per barrel of bitumen, and also eradicate any possible need for the storage of tailings in fluid form, reducing the time elapsed between the mining and reclamation processes.^9^ Syncrude recently built 18 plant‐size centrifuges, costing ≈$1.9 billion, for the purpose of MFT densification.^10^ All the mentioned technologies have one thing in common: they all use polymer flocculant to aggregate fines in tailings.

The industry standard flocculant is ultrahigh molecular weight (>10 million g mol^−1^) anionic polyacrylamide (PAM), but the sediments from PAM‐induced flocculation are loosely packed and have a gel‐like structure because they trap water in their matrix via hydrogen bonding among polymer chains and water molecules.^4^, ^11^ If the sediments' shear strength is too low, the tailings ponds will not be reclaimable, since it is not possible to build or walk on top of sediments that have weak mechanical properties. Adding comonomers with different functional groups to PAM may allow for the optimization of the copolymer properties in order to achieve better flocculation results. Some previous investigations have shown that copolymerizing acrylamide with hydrophobic monomers lowers the content of water retained in the flocs, and therefore increases the solids content of the sediments.^5^, ^6^, ^12^, ^13^

Though some research groups have tested acrylamide copolymers as flocculants for oil sands tailings, to the best of our knowledge, there are only few studies that have performed a systematic study of the causes for improved dewatering and consolidation of oil sands tailings in the open literature.^5^, ^6^, ^13^, ^14^, ^15^ For example, Reis et al. varied the copolymer composition and molecular weight of a polyacrylamide‐graft‐poly(propylene oxide) (PAM‐*g*‐PPO) copolymer, but only compared the data points in terms of flocculation performance, and did not inquire into the reason for differing results at different copolymer compositions.^5^ They compared two different lengths of the poly(propylene oxide) (PPO) grafts, using either 300 g mol^−1^ or 1000 g mol^−1^ macromonomers, but with only two different values it is not possible to determine a correlation between graft size and flocculation/dewatering performance. Their results did show that the PPO graft with the lowest molecular weight (300 g mol^−1^) achieved better results, such as lower turbidity and faster settling rate, which was a unique outcome, since most tests ascertain the opposite: higher molecular weight polymers usually performs better than lower molecular weight ones.^16^, ^17^, ^18^ The authors attributed this effect to the higher hydrophobicity of the PPO grafts. A relationship between different copolymer compositions and hydrophobic group size was not obtained, as they instead focused on optimizing the dosage and performance of the most promising PAM‐*g*‐PPO flocculant. Ren et al. flocculated kaolin suspensions with PAM, poly(diallyldimethylammonium chloride) (PDADMAC), and 3‐acrylamido‐2‐hydroxypropyltrialkylammonium chloride copolymers, where the alkyl group was ethyl, butyl, or octyl.^14^ They noticed that the hydrophobic groups enhanced dewatering ability and reduced turbidity, in comparison to PAM and PDADMAC. Ren et al. stated that the hydrophobically modified polymers were likely able to form bridges with the suspended kaolin particles, since the long alkyl chains might adsorb onto more sites on the particles.^14^ Indeed, most researchers agree that a better bridge between the polymer and suspended solids will lead to stronger flocs and better flocculation performance. Ren et al. observed that the addition of hydrophobic groups onto an acrylamide backbone increases settling rates and decreases both water retention and supernatant turbidity. Although the effect of hydrophobic chains on the PAM backbone has been recognized,^5^, ^12^ we have been unable to find a study assessing the relationship between the size of hydrophobic chain, amount of hydrophobic chain in the copolymer, polymer molecular weight, and its performance on MFT flocculation and dewatering

Laboratory scale screening of polymer flocculants for oil sands tailings is still controversial, mainly because of the lack of clear procedures and accepted values to quantify flocculation and dewatering, as well as different performance metrics required by different dewatering technologies. For example, a high settling rate of 20 m h^−1^, sought in gravity thickeners, may not be as important if dewatering is done by postflocculation centrifugation or by thin lift drying. The Alberta Energy Regulator, however, has approved Directive 085 for fluid tailings management for oil sands mining projects, where it states some minimum acceptable criteria for treated tailings before they are ready to be reclaimed (such as minimum undrained shear strength, solids content, and sand to fines ratio), which is not helpful for flocculation performance characterization.^19^ In the open literature, researchers use different performance indicators to characterize their flocculants and flocculation process for oil sands tailings, most of which were adapted from wastewater treatment and other mining applications. Among them are floc‐hindered settling rate, capillary suction time (CST), specific resistant to filtration, turbidity of supernatant, fine residues in the supernatant, shear strength of the sediment, gravity dewatering by sieve test, yield strength of sediment by slump test, solid content after centrifugation, and solid content after 24 h jar settling.^4^, ^5^, ^6^, ^20^, ^21^, ^22^, ^23^, ^24^, ^25^

In this study, we used a central composite rotatable design of experiment to analyze our results and evaluated the performance of our novel copolymers through CST, supernatant turbidity, initial settling rate (ISR), and solid content of sediments. To observe the effect of centrifugation on flocculation performance, we also tested the turbidity, solid content, and CST after centrifugation. By studying the cause of this increased flocculation performance, we will be able to understand the reason for improvement and tailor polymers for optimal performance during oil sand tailing flocculation and dewatering. It is worth noting that the intention of this study was not to optimize the proposed flocculant composition for what are arbitrarily selected conditions or a particular MFT concentration, but rather to study how certain polymer microstructure properties (MW, macromonomer size, comonomer composition) affected their performance in small‐scale tests under controlled conditions.

## 2 Results and Discussion

### 2.1 MFT Composition

A Dean Stark apparatus was used to determine the amount of solids, water, and bitumen in the tailings we used for testing. The Dean Stark apparatus comprises of a trap, round bottom flask, and reflux condenser.^5^ To begin the analysis, 140 g of MFT was placed into the trap, and 200 mL of toluene was added into the round bottom flask, where it was heated up to its boiling point. Once boiling, the vapors, consisting of water and toluene, travelled to the condenser where they condensed into the trap. Since the liquids are immiscible, the toluene, less dense than water, separated on top, and water on the bottom. Once the water level had stabilized, the water was drained and the weight of water inside of the MFT was measured. Since bitumen and toluene were mixed together, we evaporated the toluene to determine the amount of toluene and bitumen. After the process was completed, the amount of solids left from the beginning were dried and weighed to obtain the solid content of the sample. We performed this process twice to ensure accuracy of results. In addition to the Dean Stark analysis, we measured the major ion concentrations through atomic absorption spectroscopy using a VARIAN 220FS Atomic Absorption Spectrophotometer. The concentration of certain ions may impact the performance of a flocculant, so it is beneficial to know the ionic concentrations and compare with the findings.^1^ **Table** **1** lists the composition of MFT used in this work.

**Table 1:** MFT and process water composition

MFT sample		Run 1	Run 2
Dean Stark analysis (wt%)	Water	59.8	59.02
	Bitumen	2.90	3.67
	Solids	35.20	35.40
Major ion concentrations (ppm)	Na^+^	248.30	
	K^+^	18.40	
	Mg^2+^	11.00	
	Ca^2+^	22.10	

### 2.2 Experimental Results

**Figure 1:**
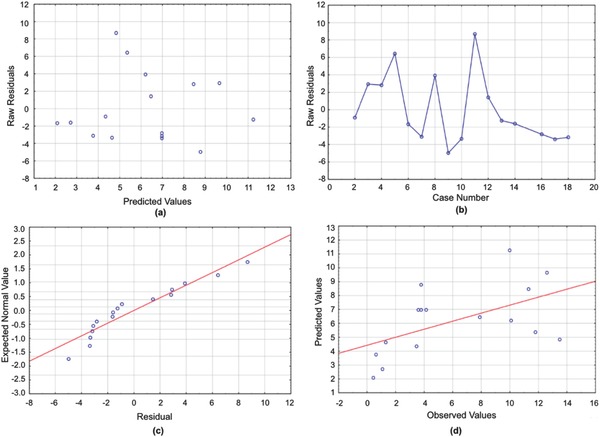
a) Residuals versus predicted values for the ISR model. b) Run sequence plot for the ISR model. c) Normal plot for the ISR model. d) Predicted versus observed values for the ISR model.

**Figure 2:**
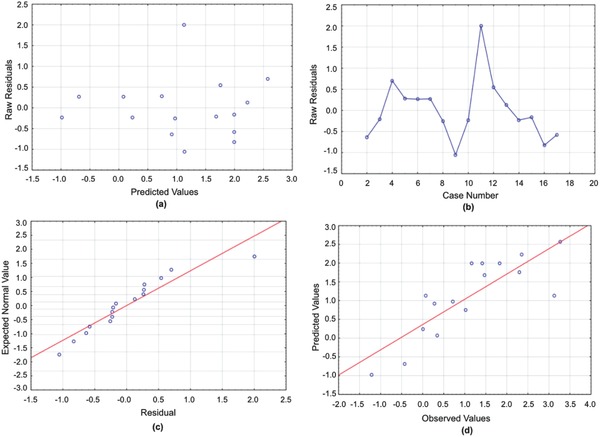
a) Residuals versus predicted values for the Turbidity model. b) Run sequence plot for the Turbidity model. c) Normal plot for the Turbidity model. d) Predicted versus observed values for the Turbidity model.

**Figure 3:**
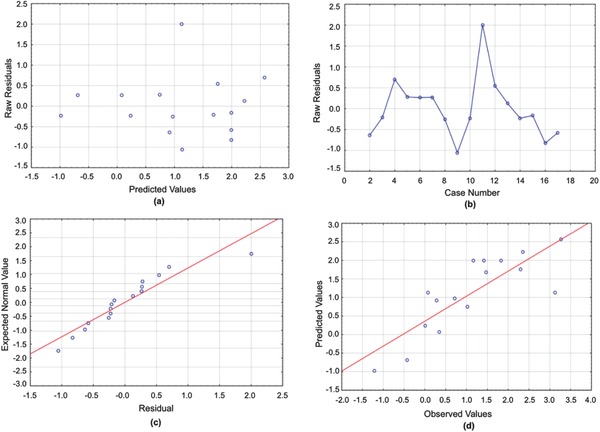
a) Residuals versus predicted values for the CST model. b) Run sequence plot for the CST model. c) Normal plot for the CST model. d) Predicted versus observed values for the CST model.

**Figure 4:**
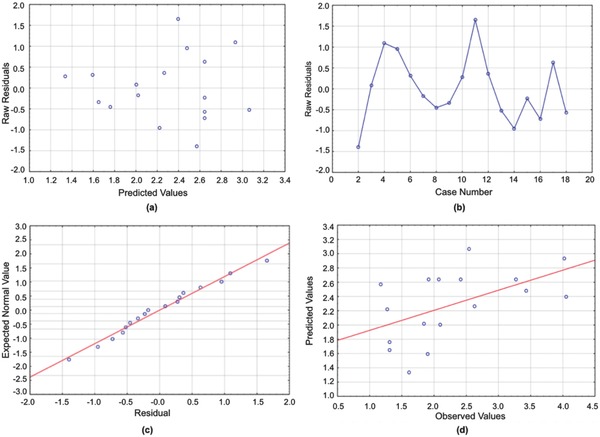
a) Residuals versus predicted values for the Centrifuge Turbidity model. b) Run sequence plot for the Centrifuge Turbidity model. c) Normal plot for the Centrifuge Turbidity model. d) Predicted versus observed values for the Centrifuge Turbidity model.

**Figure 5:**
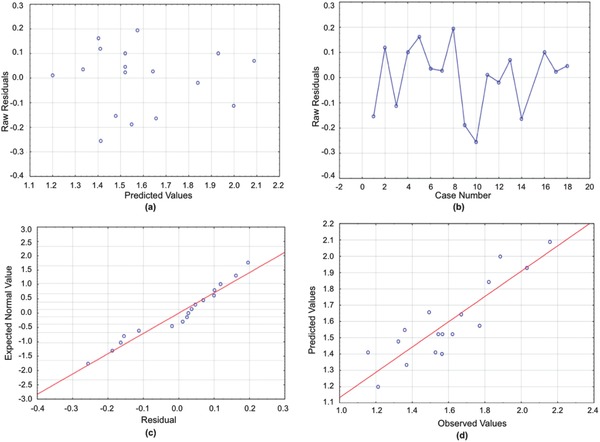
a) Residuals versus predicted values for the Solid Content Increase model. b) Run sequence plot for the Solid Content Increase model. c) Normal plot for the Solid Content Increase model. d) Predicted versus observed values for the Solid Content Increase model.

All normal and residual plots are displayed in the **Figures** **1**, **2**, **3**, **4**, **5**. In this paper, we will classify PEOMA by length only, and when “polymer molecular weight” is referred to, we refer to the molecular weight of the entire copolymer. This could be a source of confusion as the PEOMA length influences the copolymer molecular weight, but they are two separate entities.

In the ANOVA tables, we compared the three main variables, both linear (*L*) and quadratic (*Q*), and their two factor interaction effects, where the notation used is linear effect by linear effect (for example, 1*L* by 2*L* denotes the linear interaction between PEOMA length (1) and PEOMA wt% (2).

**Table** **2** lists the properties of the synthesized PAM‐*g*‐PEOMA flocculants and all observed MFT flocculation/dewatering results. A wide range of polymer properties were considered to obtain useful correlations between polymer structure and flocculation/dewatering performance.

**Table 2:** PAM‐*g*‐PEOMA properties and MFT flocculation/dewatering data

Test number	PEOMA length [*n*]	Wt% PEOMA	Log[*I*]	[*I*] [mol L^−1^]	Turbidity [NTU]	CST [s]	24 h solid content	ISR [m h^−1^]	*M*_n_ [kg mol^−1^]	PDIa)
1	9.1	14.12	−4	1.0E‐04	5.44	71.1	16.07	0.092	40	2.40
2	31.8	14.12	−4	1.0E‐04	1.32	13.1	19.56	0.376	36	2.46
3	9.1	40.88	−4	1.0E‐04	4.33	5.7	18.76	1.375	76	3.38
4	31.8	40.88	−4	1.0E‐04	26.40	7.3	16.61	1.233	121	2.70
5	9.1	14.12	−2	1.0E‐02	2.79	8.3	20.00	1.288	23	4.24
6	31.8	14.12	−2	1.0E‐02	0.65	22.8	16.93	0.049	14	2.81
7	9.1	40.88	−2	1.0E‐02	1.42	20.5	17.58	0.070	33	2.95
8	31.8	40.88	−2	1.0E‐02	2.04	7.1	17.19	1.102	21	3.95
9	1.0	27.50	−3	1.0E‐03	1.08	14.8	20.25	0.414	40	2.21
10	43.1	27.50	−3	1.0E‐03	1.01	42.1	17.31	0.141	38	2.74
11	19.3	5.00	−3	1.0E‐03	22.80	5.5	33.70	1.474	122	2.15
12	19.3	50.00	−3	1.0E‐03	10.00	9.0	18.45	0.862	1,473	2.44
13	19.3	27.50	−5	1.0E‐05	10.50	6.7	16.45	1.092	211	2.17
14	19.3	27.50	−1	1.0E‐01	0.30	18.9	18.81	0.120	12	3.39
15	19.3	27.50	−3	1.0E‐03	6.23	9.5	19.09	0.567	79	2.91
16	19.3	27.50	−3	1.0E‐03	3.21	9.2	20.01	0.453	68	3.51
17	19.3	27.50	−3	1.0E‐03	4.12	16.1	19.58	0.393	82	3.04
18	19.3	27.50	−3	1.0E‐03	0.30	13.0	19.32	0.414	59	2.97

^a)^ PDI = polymer polydispersity index.

### 2.3 PAM‐*g*‐PEOMA Molecular Weight Measurements

The molecular weight distribution of all PAM‐*g*‐PEOMA flocculants was measured by gel permeation chromatography (GPC), and the results are displayed in Table 2. Unsurprisingly, the initiator concentration, [*I*], significantly affected the molecular weight of all PAM‐*g*‐PEOMA copolymers, as it is known that the molecular weight of a polymer is controlled by the initiator concentration, and according to Shawki and Hamielec

(1) $$\frac{1}{\bar{r_{n}}}\;=\frac{\frac{k_{t}^{0.5}}{k_{p}}{\left(2fk_{I}{\left[I\right]}_{0}\right)}^{0.5}}{{\left[M\right]}_{0}\left(1-x_{n}\right)}\;+\;\frac{k_{tr}^{m}}{k_{p}}$$

where ${\bar{r}}_{n}$ represents the number‐average chain length of the copolymer, *f* is the fractional initiator efficiency, $k_{tr}^{m},\;k_{p},\;k_{I},\;\mathrm{and}\;k_{t}$ are the reaction rate constants for transfer to monomer, propagation, initiator decomposition, and termination, respectively, and [*M*] is the monomer concentration.^26^

**Figure 6:**
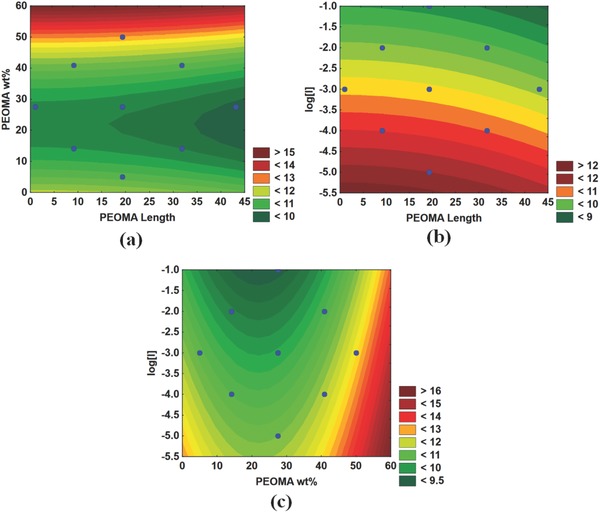
Effect of polymerization conditions on the molecular weight (*M*_n_) of PAM‐*g*‐PEOMA: a) *L*_n_(*M*_n_) versus PEOMA wt% and length, b) *L*_n_(*M*_n_) versus Log[*I*] and PEOMA length, and c) *L*_n_(*M*_n_) versus Log[I] and PEOMA wt%. Legends with distinct colors indicate the range of response (here *L*_n_(*M*_n_)).

Interestingly, PEOMA wt% in the copolymer also affected the molecular weight of PAM‐*g*‐PEOMA. **Figure** **6**a shows that the molecular weight of PAM‐*g*‐PEOMA increases as the PEOMA wt% increases, irrespective of the PEOMA length. The effect of initiator concentration on PAM‐*g*‐PEOMA molecular weight is observed in Figure 6b, where lowering [*I*] increases PAM‐*g*‐PEOMA molecular weight, as expected for solution free‐radical polymerization. Figure 6c combines the effects of [*I*] and PEOMA wt%, and shows that polymerization with low [*I*] and high PEOMA wt% will produce PAM‐*g*‐PEOMA with the highest molecular weights.

There seems to be a trend indicating a slight decrease in PAM‐*g*‐PEOMA molecular weight as the length of the PEOMA grafts increases, as shown in Figure 6b. This trend is in agreement with the results obtained by Xiao et al., where they determined the reactivity ratios of PAM/PEOMA system and discovered that the reactivity of the macromonomer (PEOMA) decreased as its chain length increased.^27^ The lower reactivity of longer PEOMA macromonomers would then lead to a lower overall copolymer molecular weight, and our findings support their conclusions. These findings do not imply that long PEOMA macromonomers cannot be used to make PAM‐*g*‐PEOMA copolymers with higher molecular weights; it only means that when all other variables are kept constant—namely initiator concentration, monomer/macromonomer concentration, and temperature—the molecular weight of PAM‐*g*‐PEOMA will decrease as the length of the hydrophobic PEOMA macromonomer increases. One could, evidently, change the polymerization conditions (such as decreasing [*I*]) to compensate for this effect.

As seen in Table 2, we produced PAM‐*g*‐PEOMA copolymers with molecular weights ranging from 12 000 to 1 473 000 g mol^−1^. This wide molecular weight range will help show the effect of PAM‐*g*‐PEOMA molecular weight on flocculation/dewatering performance. Compared with most flocculants, the molecular weights of our PAM‐*g*‐PEOMA flocculants are relatively low, as it is generally accepted that high molecular weight polymers are more effective flocculants (i.e., in terms of settling rate),^28^, ^29^, ^30^ but our recent results suggested that higher the molecular weight does not necessarily mean better dewatering in the sediments they generate, as indicated by the independence of slurry CST on MW of the cationic copolymer of acrylamide and DADAMC.^20^

### 2.4 ISR

**Table** **3** displays the ANOVA results for these analyses. The ANOVA table shows that all three manipulated variables, PEOMA length, PEOMA wt%, and log[*I*], are significant factors, and the residual plots indicate a normal distribution.

**Table 3:** ANOVA results for ISR

Factor	SSa)	dfb)	MSc)	*F* d)	*p* e)	Significance level
(1) PEOMA Length (*L*)	23.40	1	23.40	3.29	0.120	Significant
PEOMA Length (*Q*)	0.04	1	0.04	0.01	0.941	
(2) PEOMA wt% (*L*)	1.25	1	1.25	0.18	0.689	
PEOMA wt% (*Q*)	83.90	1	83.90	11.79	0.014	Significant
(3) log[*I*] (*L*)	76.18	1	76.18	10.71	0.017	Significant
Log[*I*] (*Q*)	7.77	1	7.77	1.09	0.336	
1*L* by 2*L*	104.51	1	104.51	14.69	0.009	Significant
1*L* by 3*L*	14.19	1	14.19	1.99	0.200	Significant
2*L* by 3*L*	2.92	1	2.92	0.41	0.550	
Error	42.68	6	7.11			
Total SS	328.08	15				

^a)^ SS is the sum of squares

^b)^ df is degrees of freedom

^c)^ MS is mean square

^d)^ *F* is f‐statistic

^e)^ And *p* is *p*‐value.

**Figure 7:**
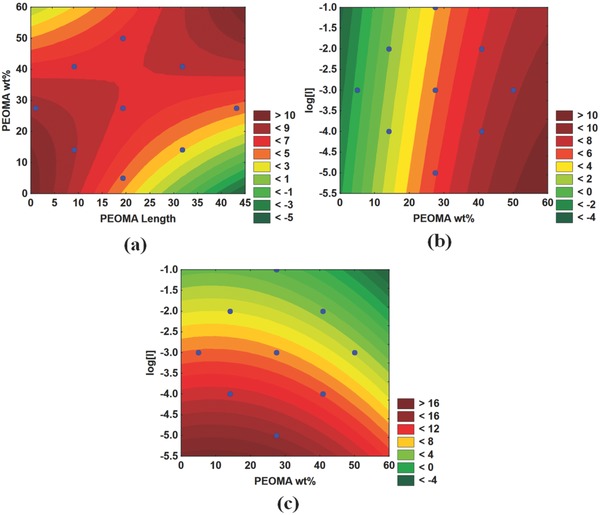
Effect of polymerization conditions on the ISR of PAM‐*g*‐PEOMA: a) ISR versus PEOMA wt% and length, b) ISR versus Log[*I*] and PEOMA wt% at a PEOMA length of 40, and c) ISR versus Log[*I*] and PEOMA wt% at a PEOMA length of 5.

Once the insignificant factors are eliminated, we can create surface plots on which the effects of the manipulated variables on ISR are depicted as a function of two variables. **Figure** **7**a shows that PAM‐*g*‐PEOMA performs best (high ISR) when the PEOMA length and wt% are high (upper right quadrant), or when the PEOMA lengths and PEOMA wt% are low (lower left quadrant). Combining this finding with the results shown in Figure 7b,c, we conclude that all PAM‐*g*‐PEOMA copolymers lead to higher ISR when their molecular weights are higher. This agrees with our knowledge of the flocculation mechanism with these polymers, as higher molecular weight PAM‐*g*‐PEOMA can create larger and heavier flocs that settle faster via the bridging mechanism. Since these polymer flocculants are neutral, charge patch and charge neutralization cannot be the underlying flocculation mechanism.

Analyzing the numbers associated with the colors in Figure 7b,c, we observe that the ISR for the PAM‐*g*‐PEOMA with low PEOMA length and low PEOMA% is almost twice as high as for the PAM‐*g*‐PEOMA with high PEOMA length and high PEOMA%. Steric hindrance of the chains may explain these results: High molecular weight PAM‐*g*‐PEOMA with long hydrophobic PEOMA chains may become very large in solution, interact with one another, and hinder the entrapment of clay particles in the MFT suspension.

Figure 7b displays the ISR as a function of initiator concentration and PEOMA wt% for PEOMA length of 40, clearly indicating that high settling rates are achieved when the molecular weight of PAM‐*g*‐PEOMA is high, and it contains high PEOMA%. The high PAM‐*g*‐PEOMA molecular weight and PEOMA wt% allow the flocculant to form strong bridges with multiple points of contact among the suspended particles, forming large, strong flocs that quickly settle.

Figure 7c displays a similar relationship for a PEOMA length of 5. The correlation between PAM‐*g*‐PEOMA molecular weight and ISR is stronger than for the longer PEO unit discussed in Figure 7b. The figure shows that ISR decreases drastically once [*I*] is lowered below 0.001 mol L^−1^. On the other hand, the weight percent of PEOMA is not as significant, as one may achieve a high ISR for any PEOMA% when the molecular weight of PAM‐*g*‐PEOMA is high (that is, when [*I*] is low), although lower PEOMA% seems to be produce slightly higher ISR values.

### 2.5 Turbidity of Supernatant

Similar to the ISR, the analysis of variance for turbidity values found out that all variables were significant, but we had to take the natural logarithm of the turbidity values in order to obtain a correlation with accurate prediction power. The ANOVA table shown in **Table** **4** lists all significant variables, and the residual plots shown in the Figures 1, 2, 3, 4, 5 yield normal distributions, confirming there is a true relationship between the variables and responses.

**Table 4:** ANOVA results for 24 h turbidity

Factor	SSa)	dfb)	MSc)	*F* d)	*p* e)	Significance Level
(1) PEOMA Length (*L*)	0.033	1	0.033	0.096	0.767	
PEOMA Length (*Q*)	2.404	1	2.404	7.016	0.038	Significant
(2) PEOMA wt% (*L*)	0.151	1	0.151	0.439	0.532	
PEOMA wt% (*Q*)	1.843	1	1.843	5.380	0.059	Significant
(3) log[I] (*L*)	9.191	1	9.191	26.82	0.002	Significant
Log[I] (*Q*)	1.049	1	1.049	3.060	0.130	Significant
1*L* by 2*L*	3.367	1	3.367	8.830	0.020	Significant
1*L* by 3*L*	0.046	1	0.046	0.136	0.725	
2*L* by 3*L*	0.237	1	0.237	0.693	0.437	
Error	2.056	6	0.343			
Total SS	23.903	15				

^a)^ SS is the sum of squares

^b)^ df is degrees of freedom

^c)^ MS is mean square

^d)^ *F* is f‐statistic

^e)^ And *p* is *p*‐value.

**Figure 8:**
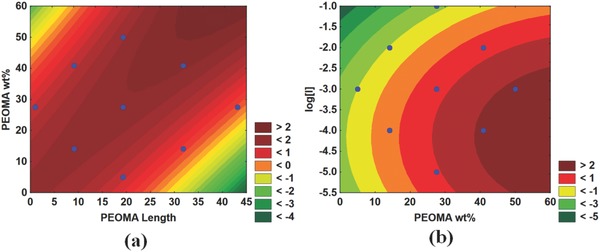
Effect of polymerization conditions on the turbidity of PAM‐*g*‐PEOMA: a) ln(turbidity) versus PEOMA wt% and length and b) ln(turbidity) versus Log[*I*] and PEOMA wt% at a constant PEOMA length of 40.

**Figure** **8**a shows that the supernatant reaches the lowest turbidities in two cases: (1) low PEOMA length and high PEOMA wt% (left upper corner), and (2) high PEOMA length and low PEOMA wt% (right lower corner). The poor results (high turbidity) obtained when PAM‐*g*‐PEOMA had a high PEOMA wt% with high length (right upper corner) may be due to steric hindrance among the long hydrophobic PEOMA grafts that lower the performance of flocculation when there is a high amount of PEOMA in the copolymer. These chains may interfere with each other and are unable to trap the finest particles. On the other hand, PAM‐*g*‐PEOMA with shorter PEOMA grafts requires a higher PEOMA% on the copolymer to trap all of the fine particles because the PEOMA chains are shorter. Having more frequent, shorter PEOMA side chains increase the surface area of PAM‐*g*‐PEOMA and create more sites onto which the flocculant may adsorb to the surface of the suspended MFT particles.

Figure 8b illustrates the effect of [*I*] and PEOMA wt% for a constant PEOMA length of 40 and confirms our conclusions above: PAM‐*g*‐PEOMA with long PEOMA grafts produces supernatant with low turbidity when a low PEOMA% is present in the polymer so that there is no steric hindrance between the hydrophobic groups. Lowering the PAM‐*g*‐PEOMA molecular weight for a given PEOMA wt% also helps decrease the turbidity for the same reasons.

These conclusions are different from those observed for ISR, as ISR was the highest when the PAM‐*g*‐PEOMA had high PEOMA wt% and high PEOMA length, while the turbidity analysis shows that high PEOMA length and low PEOMA% are ideal. We may suggest an explanation for this observation using the proposed flocculation mechanism for these polymers: When PAM‐*g*‐PEOMA polymers trap particles to form flocs, the larger flocs are heavier, and thus settle faster, but these polymers may not be able to trap all the fine particles because the larger hydrophobic PEOMA side groups interact with each other, hindering the ability to adsorb on to the suspended particles. Such copolymers would yield high ISR, but would also produce supernatants with higher turbidity. Notice that this is a discussion relative to the other PAM‐*g*‐PEOMA tested in this investigation. The highest turbidity measured with all copolymers was 26 NTU, which is still an acceptable value, even for the poorest performance among the PAM‐*g*‐PEOMA listed in Table 2.

### 2.6 CST

CST data were transformed by taking the natural logarithm of the numerical data to achieve a normal residual plot (see Figure 3) and a high correlation. Similar to the previous response variables, we constructed an ANOVA table (**Table** **5**) to show the significance of the manipulated variables on CST.

**Table 5:** ANOVA results for CST

Factor	SSa)	dfb)	MSc)	*F* d)	*p* e)	Significance level
(1) PEOMA Length (*L*)	0.522	1	0.522	5.59	0.056	Significant
PEOMA Length (*Q*)	0.630	1	0.630	6.75	0.041	Significant
(2) PEOMA wt% (*L*)	0.006	1	0.006	0.06	0.809	
PEOMA wt% (*Q*)	0.369	1	0.369	3.96	0.094	Significant
(3) log[I] (*L*)	1.194	1	1.194	12.79	0.012	Significant
Log[I] (*Q*)	0.0002	1	0.0002	0.002	0.963	
1*L* by 2*L*	1.027	1	1.027	10.99	0.016	Significant
1*L* by 3*L*	0.266	1	0.266	2.85	0.142	Significant
2*L* by 3*L*	0.005	1	0.005	0.061	0.813	
Error	0.560	6	0.0934			
Total SS	4.838	15				

^a)^ SS is the sum of squares

^b)^ df is degrees of freedom

^c)^ MS is mean square

^d)^ *F* is f‐statistic

^e)^ And *p* is *p*‐value.

**Figure 9:**
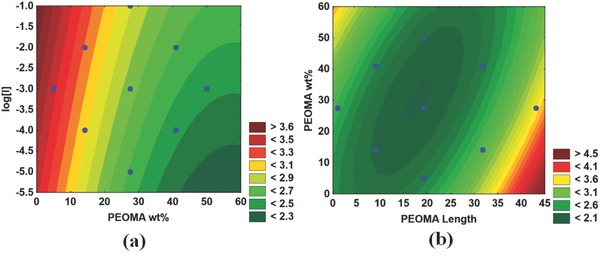
Effect of polymerization conditions on the CST of PAM‐*g*‐PEOMA: a) *L*_n_(CST) versus Log[*I*] and PEOMA wt% and b) *L*_n_(CST) versus PEOMA length and wt%.

Based on the results in Table 5, we were able to eliminate the nonsignificant variables and obtain surface plots to display the correlations among the significant variables. **Figure** **9**a shows how CST depends on [*I*] and PEOMA wt%. CST values were the lowest (fast dewatering) when PAM‐*g*‐PEOMA had a high molecular weight and PEOMA wt%. These results are easy to explain because the higher the PEOMA%, the higher the hydrophobicity of the copolymer; since CST measures the dewatering ability of flocs, a more hydrophobic flocculant is expected to retain less water inside the flocs. By the same token, a higher molecular weight PAM‐*g*‐PEOMA flocculant makes larger, more compact flocs that retain less water, and yield a lower CST. Although the effect of copolymer molecular weight on CST is not very significant, it contradicts the findings of our recent work where the CST of flocs produced by cationic copolymers of acrylamide and DADMAC did not depend on molecular weight.^20^ We believe this discrepancy is due to the fact that the dewaterability of flocs produced by cationic flocculants is so strongly affected by charge density (copolymer composition) that the little effect of molecular weight was masked.

Figure 9b plots the CST as a function of PEOMA% and length, where the green colored areas near the center of the plot indicate desirable low CST. This trend is almost the exact opposite of that observed for turbidity, and most likely occurs because of the combination of high PEOMA length and PEOMA% produces a more hydrophobic flocculant, which retains less water. Comparing Figure 9b with Figure 7a, we notice that the graphs overlap in similar fashion, with red indicating high ISR and green low CST, meaning that a satisfactory performance may be achieved in both tests by the same copolymer. Although not all tests are satisfied by the same flocculant, it shows that there is no “one size fits all” flocculant for MFT suspensions.

### 2.7 Centrifuge Turbidity

**Figure 10:**
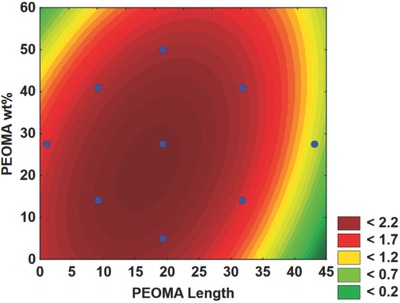
Effect of polymerization conditions on the turbidity after centrifugation of PAM‐*g*‐PEOMA flocculated MFT.

The supernatant turbidity following centrifugation yields very similar results to the turbidity after 24 h of settling. **Figure** **10** shows that PAM‐*g*‐PEOMA with long PEOMA side chains requires low PEOMA% to capture all of the fine particles, and that PAM‐*g*‐PEOMA with shorter PEOMA grafts needs higher PEOMA% in the copolymer to produce clear supernatants and follows the same pattern for the turbidity without centrifugation—the copolymers with a large percentage of long PEOMA grafts may hinder the entrapment of smaller particles, while the copolymers with a low percentage of short PEOMA grafts are unable to adsorb to all of the particles as there are fewer sites to form a proper bridge. Although we could obtain a strong correlation among these variables, it should be noted that the highest turbidity after centrifugation was only 16.4 NTU, which still corresponds to a very clear supernatant. For all practical purposes, after centrifugation, all PAM‐*g*‐PEOMA flocculants produced supernatants that were essentially free of fine particles. All polymers produced supernatants with very low turbidity. Although the differences are statistically significant and indicate that the polymer microstructure affects turbidity, these differences may have little practical significance, since all of them satisfy the condition of less than 0.5% solid residues in the supernatant indicated by Directive 085.

### 2.8 Further Consolidation through Centrifugation

An interesting observation obtained through this study was the effect of centrifugation on the solids content of the sediments, compared that obtained in settling over a single day. By dividing the solid content of the centrifuged solids by that of the 24 h settled solids, we were able to compare the increase in solid content by centrifugation. **Table** **6** reports the analysis of variance for these experiments.

**Table 6:** ANOVA results increase in solids content due to centrifugation

Factor	SSa)	df b)	MSc)	*F* d)	*p* e)	Significance level
(1) PEOMA Length (*L*)	0.0003	1	0.0003	0.01	0.915	
PEOMA Length (*Q*)	0.106	1	0.106	5.11	0.058	Significant
(2) PEOMA wt% (*L*)	0.499	1	0.499	24.16	0.002	Significant
PEOMA wt% (*Q*)	0.0003	1	0.0003	0.001	0.973	
(3) log[I] (*L*)	0.194	1	0.194	9.39	0.018	Significant
Log[I] (*Q*)	0.098	1	0.098	4.75	0.066	Significant
1*L* by 2*L*	0.007	1	0.007	0.35	0.574	
1*L* by 3*L*	0.019	1	0.019	0.95	0.362	
2*L* by 3*L*	0.039	1	0.039	1.87	0.213	Significant
Error	0.145	7	0.021			
Total SS	1.217	15				

^a)^ SS is the sum of squares

^b)^ df is degrees of freedom

^c)^ MS is mean square

^d)^ *F* is f‐statistic

^e)^ And *p* is *p*‐value.

**Figure 11:**
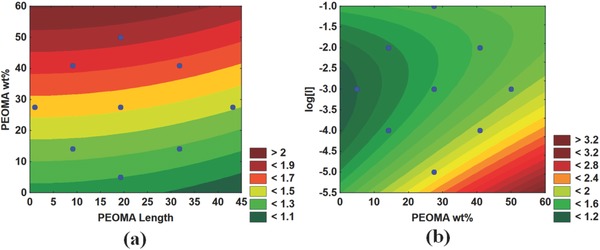
Effect of polymerization conditions on the solid content increase of PAM‐*g*‐PEOMA: a) solid content increase versus PEOMA length wt% and b) solid content increase versus Log[*I*] and PEOMA wt%.

The red areas in **Figure** **11**a,b display the polymers for which the solid content increased the most by centrifugation. The higher the PEOMA% in PAM‐*g*‐PEOMA, the greater the increase in solids content. The centrifuge increases the g‐force on the sample, forcing the flocs to settle to the bottom of the centrifuge tubes, where the high hydrophobic PEOMA groups decrease the amount of water trapped inside of the sediments. Equally important, the lower the [*I*], the higher the molecular weight of PAM‐*g*‐PEOMA, and the greater the effect of centrifugation on the solids content. The impact of PAM‐*g*‐PEOMA molecular weight is shown most clearly in Figure 11b, as the area of the graph showing the greatest increase in solids content corresponds to low [*I*] values and high PEOMA%. As shown by Figure 11a, PEOMA length is not very significant in terms of solids content improvement by centrifugation. This may be because the PEOMA length was not significant in the molecular weight of the copolymer, and thus, the additional g‐force does not improve compaction of the sediments based on this variable.

### 2.9 Applications of Our Model to Other Systems

As mentioned in the introduction, Reis et al. explored the addition of hydrophobic groups to an acrylamide backbone. They found similar results with the PAM‐*g*‐PPO copolymer, where the shorter length hydrophobic grafts combined with a high percentage of grafts performed the best.^5^ Also, they found that one of the best copolymers using the longer PPO length (1000 g mol^−1^ macromonomer) was when they used only 5 wt% PPO. The combination of a long hydrophobic chain length and low wt% was required in our model so there was no steric hindrance. The only copolymer with the longer PPO length that performed better in some tests was a 35 wt% PPO, but it performed better in CST, where our model states that the additional hydrophobic groups would expel water faster, producing a lower value. The alignment between their results and our conclusions confirms that our model is applicable in other polymer systems where a hydrophobic chain is added to acrylamide.

Shang et al. also added hydrophobic groups to acrylamide, making poly(acrylamide‐methacryloxyethyl trimethyl ammonium chloride‐methacryloxypropyltrymethoxy silane) (P(AM‐DMC‐MAPMS)).^13^ The hydrophobic groups are relatively small, only having two and three repeating units for the DMC and MAPMS, respectively. According to our model, the shorter hydrophobic group length would indicate that a larger amount of the DMC and MAPMS would be required in order to achieve optimal effects. Although their flocculation testing was not performed using MFT, they still concluded that the higher feed ratios of hydrophobic group increased the performance of the flocculant. Their findings also coincide with ours in the ISR and Turbidity sections, as the low chain length required a high molecular weight, represented by intrinsic viscosity in their paper, and a high percentage of hydrophobic groups to perform well. Again, our model has shown to be pertinent to other copolymer systems involving hydrophobic modifications to acrylamide.

## 3 Conclusion

As displayed through the different optimal variables for each one of the tests, there is no “perfect” polymer for treating MFT; however, it is possible to tailor a polymer for specific flocculation purposes. Depending on the required characteristics of flocculation, one is able to synthesize a polymer based on the criteria. This study allowed us to delve deeper into the reasons behind enhanced flocculation performance gained by the addition of hydrophobic chains to an acrylamide backbone. Through the analysis of the effects of initiator concentration, PEOMA length, and PEOMA wt%, we determined the effect of hydrophobic chain addition to acrylamide polymers. Adding many hydrophobic chains (>30 wt%) can effectively help in the dewatering performance of the flocculant, shown in the ISR and CST tests, but may not capture all of the fine particles, leading to a higher turbidity. A lower initiator concentration (higher molecular weight) was also shown to improve flocculation performance in most categories. The length of hydrophobic chains was observed to be of mild importance—the shorter chains required a larger percentage of hydrophobic groups to achieve the same effects as the larger chains, whereas the longer hydrophobic chains needed a low weight percent of PEOMA to prevent steric hindrance.

Although most PAM‐*g*‐PEOMA copolymer molecular weights studied were low (<200 000 g mol^−1^), we still observed very good flocculation performances from all polymers tested. This contradicts the most common assumptions about flocculants, but it actually has some advantages as well. There are benefits to having low molecular weights, as high molecular weight flocculants will lead to an increase in viscosity and may cause problems such as nonuniform mixing. Reis et al. also noted that having a lower molecular weight flocculant allows for a wider window of mixing environments in which the flocculant may be used.^5^ One should note that the findings of this study are restricted to the conditions we investigated in our experiments and cannot be extended uncritically to other situations (such as different vessel geometries, shear rates, primary solids contents, and polymer dosages) or even other tailings. Inspired from our study, more research should be conducted to optimize the microstructure of polymer flocculants so they can achieve their optimum performances under a wide range of operating conditions.

## 4 Experimental Section

*Materials*: Monomers (acrylamide, ≥99.9% pure, and poly(ethylene glycol) methyl ether methacrylate (PEOMA)), initiator (2,2′‐Azobis(2‐methylpropionamidine) dihydrochloride (V‐50)), and organic solvents (acetone, toluene) were purchased from Sigma Aldrich. Coanda Research and Development supplied the MFT used in this investigation.

**Figure 12:**
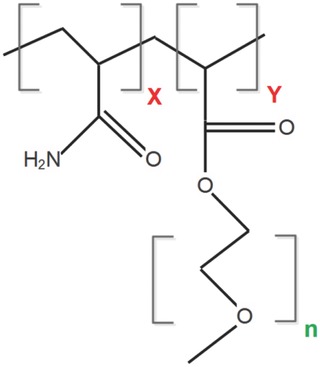
PAM‐*g*‐PEOMA copolymer structure, where “X,” “Y,” and “*n*” refer to the repeating units of the portion in parentheses.

*Polymerization*: The copolymers were synthesized by free‐radical polymerization at 40 °C in a batch reactor. The reagents were added to the reactor in the following order: deionized water, acrylamide, and then PEOMA. Once in the reactor, the solution was purged with nitrogen for 1 h to ensure a complete nitrogen atmosphere. After that, the initiator, also purged with nitrogen, was added to begin the polymerization. The polymerization was left to run for 24 h to obtain high monomer conversion for all experiments. The total monomer concentration was kept constant at 0.1 mol L^−1^ for all polymerizations. Once the reaction had finished, the polymer, whose structure is shown in **Figure** **12**, was precipitated in a 90:10 acetone/toluene mixture, rewashed in acetone for purification for at least three times, then dried for 24 h in a vacuum oven at 50 °C.

*Molecular Weight Measurement*: The molecular weights of all copolymers were measured by gel permeation chromatography using an Agilent 1260 Infinity GPC and PL aquagel‐OH Mixed‐H 8 µm columns. PEO standard samples with narrow molecular weight were used for the column calibration. The concentration of all samples was 3.6 mg mL^−1^, diluted in an aqueous solution containing 0.2 m NaNO_3_, and injected at a volume of 100 µL. The analysis was performed at a flow rate of 1 mL min^−1^ and at a temperature of 30 °C.

*Experimental Design*: Utilizing a central composite rotatable design allows us to compare the effects of macromonomer size, copolymer composition, and molecular weight as a function of flocculation performance. Macromonomer refers to a macromolecule with a functional group to allow further polymerization. As per the design, there were five different coded values for each variable, where 15 unique combinations and an additional three repetitions of the center point to ensure reproducibility were tested, allowing us to create a surface to map the effects of the design variables shown in **Table** **7**.

**Table 7:** Independent variables used for design of experiment, where PEOMA length is denoted by “*n*” (see Figure 6), wt% is g/g, and [*I*] is in mol L^−1^

	Range of values to complete design				
	−α	−1	0	+1	+α
Independent variables	−1.68	−1.00	0.00	1.00	1.68
Average PEOMA length (*n*)	1.00	9.10	19.30	31.80	43.13
Wt% PEOMA	5.00	14.12	27.50	40.88	50.00
Log([*I*])	−5.00	−4.00	−3.00	−2.00	−1.00

Although Table 7 specifies negative and positive alpha values being ±1.68, the analysis may still be performed if the true values are reasonably close to the theoretical value. As the molecular weight of the copolymer cannot be precisely controlled, the initiator concentration, [*I*], was varied to obtain a range of copolymer molecular weights. All data through Statistica 13 (StatSoft Inc.) were analyzed. In Table 7, PEOMA chain length and wt% are independent variables that can be changed without altering each other. Note that the full performance optimization of the copolymer would require testing it at different dosages, shear rates, solids contents, and water chemistries. Such an extensive study, however, would substantially add to the complexity of the process, and likely make the regression analysis unreliable. Therefore, the flocculation window by fixing these additional variables and concentrating on the impact of three main microstructural features: average PEOMA length, PEOMA composition, and copolymer average molecular weight was narrowed down.

*Test Methods*: In order to create the MFT slurry for testing, the MFT sample was diluted to 5 wt% solids in 160 g of slurry, using deionized water. The concentration of solids in the suspension to obtain as uniform mixing as possible when testing different polymers, and to maximize the ability to observe performance differences upon addition of polymers with different microstructures was diluted. The slurry was mixed at 610 rpm for 30 s to obtain a homogeneous distribution of solids in the slurry. At that point, 1000 ppm of a Ca^2+^ solution was added and mixing continued for an additional minute. The addition of calcium is necessary, as the neutral polymer flocculants here used are unable to form bridges with the suspended particles without the charge neutralization effect obtained by calcium ions. The polymer dosage was kept constant at 10 000 ppm (mg polymer per kg of dry solid) for all experiments, and the polymer mixed with the slurry at 610 rpm. The dosage of 10 000 ppm in this study was chosen based on preliminary tests with the polymers (from the highest MW to the lowest MW), and it was found out that this dosage was not too far from the working window of the polymers. It is true that 10 000 ppm would be considered a high dosage in practical application (as opposed to 1000–1500 ppm dosages used for commercial A‐PAM);^20^ however, it was not the intention of this study to optimize the polymer microstructure according to dosage.

Once flocs formed, the mixing speed was reduced to 300 rpm and stirring continued for another 2 min. After flocculation was done, while the flocculated suspension was still under gentle mixing (to achieve uniform solid concentration upon sampling), a large pipet (0.5 cm diameter) was used and separated the flocculated suspension into three different portions: (1) A volume of 10 mL was used for testing CST with a Triton Electronics Meter (Type 319 multi‐CST) with Triton filter paper (7 cm by 9 cm); (2) An aliquot of 50 mL was transferred to a Corning centrifuge tube for centrifugation purposes; and (3) The remaining 100 mL of sample was poured into a graduated cylinder so the change in mudline level could be recorded as a function of time.

After the slurry had settled in the graduated cylinder for 24 h, the supernatant was extracted for turbidity measurement in a Hach 2100 AN Turbidmeter, and additional CST measurements were performed on the solids after settling. The 5 mL of settled solids were also taken, and the solids content was calculated by weighing the sample before and after 24 h drying in an oven at 60 °C. The solids content is calculated by the following formula, where weight was measured in grams

(2) $$\mathrm{SC}\%\;\;=\;\;\frac{\mathrm{dry}\;\mathrm{weight}\;-\;\mathrm{aluminum}\;\mathrm{foil}\;\mathrm{weight}}{\mathrm{wet}\;\mathrm{weight}\;\;-\;\mathrm{aluminum}\;\mathrm{foil}\;\mathrm{weight}}\;\;\times \;\;100\%$$

## Conflict of Interest

The authors declare no conflict of interest.
